# Lower Frequency of CD62L^high^ and Higher Frequency of TNFR2^+^ Tregs Are Associated with Inflammatory Conditions in Type 1 Diabetic Patients

**DOI:** 10.1155/2011/645643

**Published:** 2011-04-10

**Authors:** Monika Ryba, Karolina Rybarczyk-Kapturska, Katarzyna Zorena, Małgorzata Myśliwiec, Jolanta Myśliwska

**Affiliations:** ^1^Department of Immunology, Medical University of Gdańsk, Dębinki 1, 80-210 Gdańsk, Poland; ^2^Diabetological Department, Clinic of Pediatrics, Hematology, Oncology and Endocrinology, Medical University of Gdańsk, 80-210 Gdańsk, Poland

## Abstract

Diabetes type 1 is a chronic autoimmune disease in which insulin-producing cells are gradually destroyed by autoreactive T cells. Human regulatory cells play important role in controlling autoimmunity, and their qualitative or quantitative dysfunctions may result in ineffective suppression of autoreactive T cells. CD62L is a surface molecule that plays role in homing capabilities of Tregs, and only cells with high expression of CD62L have high suppressive potential. Tregs are also characterized by the constant expression of TNFR2. The frequency of Tregs carrying TNFR2 is higher in inflammatory conditions. We investigated blood regulatory T cells with CD62L expression and regulatory T cells expressing TNFR2 in type 1 diabetic patients. We found differences in these populations when comparing to healthy individuals. We propose that these may be associated with inflammatory conditions that are present in patients with type 1 diabetes. The lower percentage of Tregs and Treg CD62L^high^ may contribute to ineffective suppression of proinflammatory cytokines production during type 1 diabetes.

## 1. Introduction

Diabetes type 1 is a chronic autoimmune disease that is characterized by the destruction of insulin-producing cells. It has been shown that this disorder involves failure in immune regulation [1, 2]. NOD mice and patients with type 1 diabetes have deficiencies in at least two T-cell populations with regulatory properties: NKT and CD4^+^CD25^+^ [3–5]. Human CD4^+^CD25^+^ regulatory T cells, that are characterized by high expression of CD25 molecule-comprise 5–10% of peripheral CD4^+^ T cells [6, 7] and are characterized by the upregulation of the IL-2receptor alfa-chain (CD25) and expression of the transcription factor Foxp3 [8, 9]. Some data suggest that regulatory T cells are most effective in preventing priming of naive cells [10]. This requires their presence in lymph nodes. The receptor that homes to lymph nodes is CD62L molecule (L-selectin). Lower expression of CD62L was observed on CD3^+^ lymphocytes from diabetic type 1 patients in comparison to their healthy counterparts [11]. In addition, the low percentage of CD4^+^CD25^+^CD62L^+^ and their poor efficacy in preventing autoimmunity was observed in NOD mouse [12–15]. Another surface molecule which seems to be important for regulatory T cells is the TNF receptor type 2 (TNFR2). Its constant expression was observed on Tregs [16, 17]. It was also shown that exposure to TNF enhanced the percentage of Tregs expressing TNFR2 and downmodulated their suppressive activity at the same time [17]. Chronic inflammation present in autoimmune environment may impair the number and/or function of Tregs, which then are not able to prevent the activity of inflammatory cells. As inflammation proceeds, the risk for vascular complications increases [18, 19].

Here, we demonstrate that patients with type 1 diabetes have lower percentage of peripheral blood regulatory T cells with CD62L expression and higher percentage of regulatory T cells expressing TNFR2 than healthy individuals. We propose that these differences may be associated with inflammatory conditions that are present in patients with type 1 diabetes. 

## 2. Material and Methods

### 2.1. Patients

35 patients aged 15.5 (±3.2) years with long-standing diabetes type 1 and 20 age-matched (19.8 ± 1.2 years) healthy individuals had fresh blood samples collected for analysis. The mean duration of diabetes was 6.45 (±3.7) years. The patients were recruited from The Diabetic Department, Clinic of Pediatrics, Hematology, Oncology and Endocrinology, Medical University of Gdańsk. Type 1 diabetes was defined according to the criteria of the American Diabetes Association [20]. A blood glucose level was taken at the time of sampling along with biochemical measurement of renal function, lipid status, CRP, and glycosylated hemoglobin (HbA1c). At each examination, systolic and diastolic blood pressure was measured. The study followed the principles of the Declaration of Helsinki and was approved by The Ethics Committee of The Medical University of Gdańsk. Clinical characteristics of the patients are presented in Table 1. 

### 2.2. Flow Cytometric Analysis

Blood samples (50 *μ*L) were stained with anti-CD4 (IgG1, *κ* mouse Pe/Cy5, Clone RPA-T4, BioLegend, USA), anti-CD25 (IgG1, *κ* mouse FITC, Clone BC96, BioLegend, USA), anti-TNFR2 (IgG2A mouse PE, Clone: 22235, R&D Systems Inc, USA), and anti-CD62L (IgG1, *κ* mouse APC, Clone DREG-56, BioLegend, USA) human antibodies. For each set, appropriate isotypic control was done. After 30-minute incubation in the dark at a room temperature, probes were fixed using Immunoprep reagents (Immunotech, USA) with Q-prep Immunology Workstation (Coulter, USA). 

**Figure 1:**
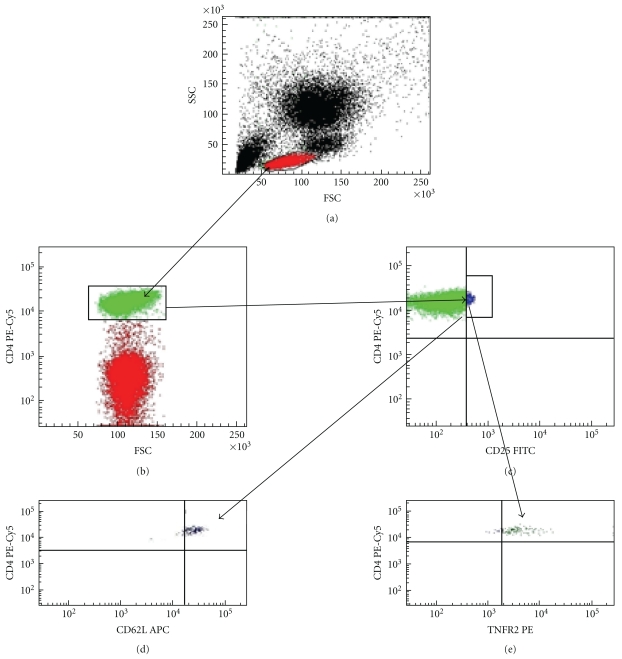
Gating strategy used to analyze CD4^+^CD25^high^ regulatory T cells in peripheral blood. The whole blood was stained with antibodies against Treg cell surface molecules. Sample gated on lymphocytes according to forward and side scatter (FSC/SSC) (a). Plots showing gate for CD4^+^ T cells (b) and CD4^+^CD25^high^ cells (c). Cells in CD4^+^CD25^high^ gate gated on TNFR2^+^ (d) and CD62L^high^ cells (e).

Expression of cell surface and intracellular markers was assessed using flow cytometry (LSRII, Becton Dickinson, USA) after gating on live cells determined by scatter characteristics. Data were analyzed by FACSDiva 6.0 Software (Becton Dickinson, USA). The expression of CD62L in the CD4^+^CD25^high^CD62L^high^ as well as expression of TNFR2 in the CD4^+^CD25^high^TNFR2^+^gates were quantified by determining mean fluorescent intensity (MFI).  Figure 1 shows gating strategy used for analysis of regulatory T-cell subsets. 

### 2.3. Determination of TNF Level

Serum level of TNF was measured by immunoenzymatic ELISA method (Quantikine High Sensitivity Human by R&D Systems Inc, USA) according to the manufacturer protocol. Minimum detectable concentrations were determined by the manufacturer as 0.12 pg/mL.

### 2.4. Statistical Analysis

All statistical analyses were performed using Statistica 8.0 (StatSoft, Inc USA). 

The differences between the groups were calculated with the nonparametric *U*-Mann Whitney tests. Multiple regression analysis was performed to determine if CD4^+^CD25^high^ T cell level depends on age and disease duration in diabetic group. Spearman's correlations were used to compare cell frequencies with analyzed parameters. *P* values less than.05 were considered statistically significant.

## 3. Results

### 3.1. Peripheral Blood CD4^+^CD25^high^, CD4^+^CD25^high^ CD62L^high^, and CD4^+^CD25^high^TNFR2^+^ T Cells

The multiple regression analysis showed no association between the peripheral blood level of CD4^+^CD25^high^ regulatory T cells and age as well as disease duration in diabetic group (*β* = [−0.04] and [−0.37], resp., *P* = .2).

Children with type 1 diabetes were characterized by lower number and percentage of the CD4^+^CD25^high^ T cells (Table 2,  *P* = .00084, *P* = .00026), lower number and percentage of the CD4^+^CD25^high^CD62L^high^ (Table 2,  *P* = .00012, *P* = .0000) than their healthy counterparts. When numbers of the CD4^+^CD25^high^TNFR2^+^ T cells were compared, the opposite effect was revealed. Tregs expressing TNFR2 were more frequent in DM1 group; however, this was true only when the percentage of cells was taken into account (Table 2,  *P* = .0000).

**Figure 2:**
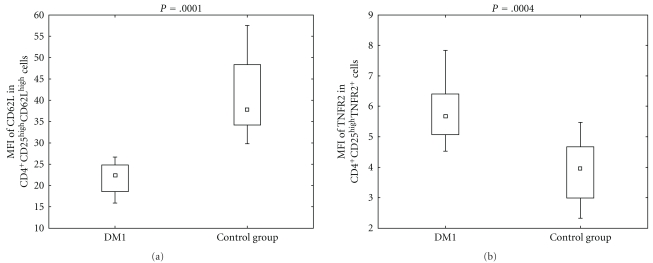
Expression of CD62L and TNFR2 defined as mean fluorescence intensity in diabetic type 1 patients and healthy individuals. During cytometric analysis of peripheral blood CD4^+^CD25^high^ T cells, the mean fluorescent intensity of CD62L and TNFR2 was defined. The MFI of CD62L in CD4^+^CD25^high^CD62L^high^ gate in diabetic group was 22.34 (15.91/26.6) and 37.7 (29.8/57.5) in control group (*P* = .0001). The MFI of TNFR2 in CD4^+^CD25^high^TNFR2^+^ gate was 5.66 (4.52/7.83) in DM1 group and 3.96 (2.32/5.37) in the control group (*P* = .0004). The data are presented as median and 10th percentile/90th percentile. The differences were calculated by *U*-Mann Whitney test.

As to the expression of CD62L and TNFR2 in CD4^+^CD25^high^CD62L^high^ and CD4^+^CD25^high^TNFR2^+^ cells, respectively, flow cytometric analysis indicated that there was a significant difference in the MFI of these molecules between analyzed groups. The expression of CD62L was higher in control group (Figure 2, *P* = .0001), while the expression of TNFR2 was higher in DM1 group (Figure 2, *P* = .0004). 

### 3.2. The Association between the Percentage of Treg Subsets and Clinical Parameters in DM1 Group

**Figure 3:**
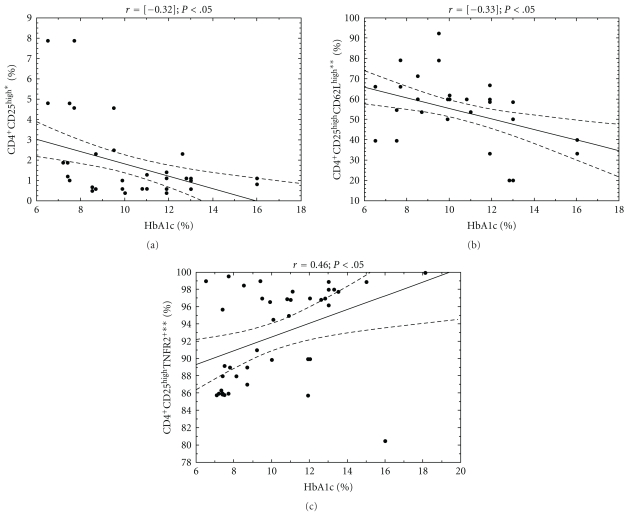
Relationship between CD4^+^CD25^high^ regulatory T-cells, their subpopulations frequencies, and HbA1c level in analyzed DM1 subjects. The level of HbA1c was measured in the blood of DM1 children and correlated with the percentage of peripheral blood regulatory T-cell subpopulations. The Spearman test was used to calculate the strength of correlation. (a) The correlation between CD4^+^CD25^high^ frequency and HbA1c level in DM1 subjects (*r* = [−0.32]); (b) the correlation between CD4^+^CD25^high^CD62L^high^ frequency and HbA1c level in DM1 subjects (*r* = [−0.33]); (c) the correlation between CD4^+^CD25^high^TNFR2^+^ frequency and HbA1c level in DM1 subjects (*r* = 0.46). *The percentage of cells among peripheral blood lymphocytes, **the percentage of cells among CD4^+^CD25^high^ lymphocytes.

**Figure 4:**
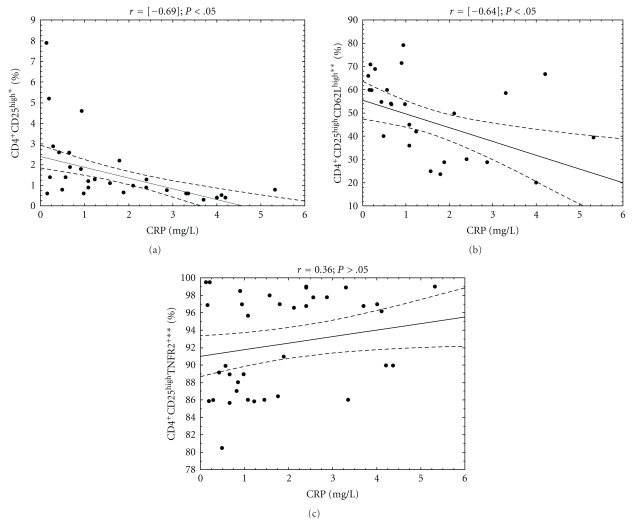
Relationship between CD4^+^CD25^high^ regulatory T cells, their subpopulations frequencies, and CRP level in analyzed DM1 subjects. The level of C-reactive protein was measured in the blood of DM1 children and correlated with the percentage of peripheral blood regulatory T-cell subpopulations. The Spearman test was used to calculate the strength of correlation. (a) The correlation between CD4^+^CD25^high^ frequency and CRP level in DM1 subjects (*r* = [−0.69]); (b) the correlation between CD4^+^CD25^high^CD62L^high^ frequency and CRP level in DM1 subjects (*r* = [−0.64]); (c) the correlation between CD4^+^CD25^high^TNFR2^+^ frequency and CRP level in DM1 subjects (*r* = 0.36). *The percentage of cells among peripheral blood lymphocytes, **the percentage of cells among CD4^+^CD25^high^ lymphocytes.

In DM1 group, the association analysis correlating HbA1c and CRP levels versus the CD4^+^CD25^high^ and their subpopulations frequency was performed (Figures 3 and 4). In the study group, the level of HbA1c was in significant relation with the frequency of CD4^+^CD25^high^ (*r* = [−0.3]; *P* < .05) CD4^+^CD25^high^CD62L^high^ (*r* = [−0.33]; *P* < .05) as well as CD4^+^CD25^high^TNFR2^+^(*r* = 0.46; *P* < .05). The significantly negative correlation was also found between CRP level and the frequency of CD4^+^CD25^high^ (*r* = [−0.69]; *P* < .05) as well as CD4^+^CD25^high^CD62L^high^ (*r* = [−0.064]; *P* < .05).

### 3.3. The Relationship between CD4^+^CD25^high^ Regulatory T-Cell Subpopulation Frequencies and Serum TNF Level in Diabetic Type 1 Children

**Figure 5:**
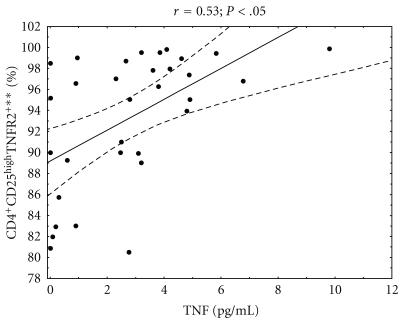
Relationship between the percentage of CD4^+^CD25^high^TNFR2^+^ regulatory T cells and serum TNF level in analyzed DM1 subjects. The level of TNF was measured in serum of diabetic type 1 patients and correlated with the percentage of peripheral blood regulatory T cells carrying TNFR2 in these patients. The Spearman test was used to calculate the strength of correlation (*r* = 0.5; *P* < .05). **The percentage of cells among CD4^+^CD25^high^ lymphocytes.

We then studied the association between serum level of TNF and the percentage of peripheral blood regulatory T cells in analyzed DM1 subjects. We found that diabetic type 1 children with higher percentage of CD4^+^CD25^high^TNFR2^+^ cells among peripheral blood CD4^+^CD25^high^ had higher serum level of TNF (Figure 5, *r* = 0.53; *P* < .05). When analyzing CD4^+^CD25^high^ and CD4^+^CD25^high^CD62L^high^ subpopulations, we did not find any association between frequency of these cells and TNF serum level. 

## 4. Discussion

Some authors have previously suggested that patients with type 1 diabetes have lower frequency of regulatory T cells in peripheral blood [5, 21]. Our work clearly revealed that children with type 1 diabetes are characterized by lower percentage and absolute number of CD4^+^CD25^high^ regulatory T cells as compared with age-matched healthy individuals. These quantitative defects may be caused by the chronic inflammation, which is observed in diabetic type 1 patients [18, 19, 22] and was observed in our study group. The analyzed patients had high serum level of CRP and detectable level of TNF, which are indicators of chronic inflammatory response [18, 19, 22]. The strong correlation was observed in our study group; patients with lower CRP level had higher percentage of CD4^+^CD25^high^ cells among peripheral blood lymphocytes than patients with higher CRP level.

Some data concerning regulatory T cells have shown that these cells are susceptible to the effects mediated by the proinflammatory cytokine—TNF [23], which may impair their activity through TNFR2 signalization [17].Our results are consistent with the findings of Valencia et al., who demonstrated that CD4^+^CD25^high^ Tregs from patients with active RA contained greater percentage of TNFR2^+^ cells compared with healthy controls [17]. Our studies gave similar results showing that the CD4^+^CD25^high^TNFR2^+^ T cells from DM1 patients had higher expression of TNFR2 than cells from the control group. Moreover, our studies revealed the correlation between percentage of CD4^+^CD25^high^TNFR2^+^ lymphocytes and serum level of HbA1c as well as CRP. Patients with poor metabolic control and higher CRP level had higher percentage of Tregs carrying TNFR2. These patients also produced more TNF than those with lower percentage of regulatory T cells expressing TNFR2. The observed greater CD4^+^CD25^high^TNFR2^+^ frequency in DM1 patients may reflect the greater sensitivity of this cell subset to inflammatory conditions and the action of TNF.

In view of the fact that in case of diabetes only subpopulation of Tregs that carries CD62L has high suppressive activity [13, 15], we can suspect that the lower percentage of CD4^+^CD25^high^CD62L^high^cells among peripheral blood Tregs as well as the expression of CD62L on CD62L^high^ Tregs in DM1 group may be associated with defect in the homing capabilities of this cell population, as CD62L is responsible for migration of Tregs to secondary lymphoid tissues [13]. In addition, when analyzing CD4^+^CD25^high^CD62L^high^ subset, we found that patients with lower percentage of these cells had higher serum CRP level, in which elevated level may reflect an inflammatory state [24]. They also had higher HbA1c level which is an indicator of metabolic control in diabetic patients [25]. Higher HbA1c is associated with higher serum TNF level [26, 27], so more intense inflammatory response. This may further cause disruption in regulatory T-cell population. The lower numbers of CD62L^high^ Treg subpopulation in type 1 diabetic patients give weight to the importance of regulatory T-cell migration to secondary lymphoid tissues in order to control activity of inflammatory cells.

The inflammatory conditions seen in patients with type 1 diabetes may modulate Treg subsets, such that they are not able to control inflammation. Further studies are needed to determine the suppressive activity of these cells in order to see if their function is disrupted.

## Figures and Tables

**Table 1:** Clinical characteristics of patients with type 1 diabetes.

Clinical parameter	Mean ± Standard deviation
Age (years)	15.5 ± 3.2
Duration of diabetes (years)	6.45 ± 3.7
BMI	19.9 ± 3.43
CRP (mg/L)	3.62 ± 1.24
HbA1c (%)	10.1 ± 2.8
Systolic blood pressure (mmHg)	116.0 ± 14.6
Diastolic blood pressure (mmHg)	74.0 ± 10.0
Serum creatinine level (mg/dL)	0.74 ± 0.15
Serum albumin level (g/L)	19.85 ± 12.16

Data are shown as mean ± standard deviation.

**Table 2:** The percentage and number of CD4^+^CD25^high^, CD4^+^CD25^high^CD62L^high^, and CD4^+^CD25^high^TNFR2^+^ T cells.

	Children with DM1	Control group	*P*
	(*n* = 35)	(*n* = 20)	
The percentage of CD4^+^CD25^high^ cells (%)*	1.56 (0.4/4.6)	3.30 (2.5/4.9)	**.00026**
The absolute number of CD4^+^CD25^high^ (cells/mm^3^)	0.37 (0.03/4.17)	1.13 (0.55/2.21)	**.00084**
The percentage of CD4^+^CD25^high^ CD62L^high^cells (%)**	54.3 (20.0/79.2)	90.2 (76.4/94.3)	**.00000**
The absolute number of CD4^+^CD25^high^ CD62L^high^ cells (cells/mm^3^)	0.014 (0.02/2.76)	1.01 (0.48/2.08)	**.000012**
The percentage of CD4^+^CD25^high^TNFR2^+^cells (%)**	96.9 (85.7/99.8)	34.95 (21.6/46.0)	**.0000**
The absolute number of CD4^+^CD25^high^TNFR2^+^cells (cells/mm^3^)	0.27 (0.03/4.158)	0.4 (0.11/1.08)	.26

Results are shown as median and 10th percentile/90th percentile. All the differences were calculated by the *U*-Mann Whitney test.

*The percentage of cells among peripheral blood lymphocytes.

**The percentage of cells among CD4^+^CD25^high^ lymphocytes.

## Abbreviations

CRP: C-reactive protein
DM1: Diabetes mellitus 1
MFI: Mean fluorescent intensity
NKT: Natural killer T
NOD: Nonobese diabetic
PBMC: Peripheral blood mononuclear cells
RA: Rhematoid arthritis
TNFR: Tumor necrosis factor receptor
Treg: Regulatory T cells.
